# The Great Gobi A Strictly Protected Area: Characterization of Soil Bacterial Communities from Four Oases

**DOI:** 10.3390/microorganisms12020320

**Published:** 2024-02-03

**Authors:** Antonia Esposito, Sara Del Duca, Francesco Vitali, Gaia Bigiotti, Stefano Mocali, Giulia Semenzato, Alessio Papini, Giacomo Santini, Nadia Mucci, Anna Padula, Claudia Greco, Battogtokh Nasanbat, Gantulga Davaakhuu, Munkhtsetseg Bazarragchaa, Francesco Riga, Claudio Augugliaro, Lorenzo Cecchi, Renato Fani, Marco Zaccaroni

**Affiliations:** 1 Council for Agricultural Research and Economics, Research Centre for Agriculture and Environment, 50125 Florence, Italy; antonia.esposito@unifi.it (A.E.); sara.delduca@crea.gov.it (S.D.D.); francesco.vitali@crea.gov.it (F.V.); gaia.bigiotti@crea.gov.it (G.B.); stefano.mocali@crea.gov.it (S.M.); 2 Department of Biology, University of Florence, Via Madonna del Piano 6, Sesto Fiorentino, 50019 Florence, Italy; giulia.semenzato@unifi.it (G.S.); alessio.papini@unifi.it (A.P.); giacomo.santini@unifi.it (G.S.); 3 Unit for Conservation Genetics (BIO-CGE), Institute for Environmental Protection and Research, Via Ca’ Fornacetta, 9, Ozzano dell’Emilia, 40064 Bologna, Italy; nadia.mucci@isprambiente.it (N.M.); anna.padula@isprambiente.it (A.P.); claudia.greco@isprambiente.it (C.G.); 4 Institute of Biology, Mongolian Academy of Sciences, Peace Avenue-54B, Bayanzurkh District, Ulaanbaatar 13330, Mongolia; battogtokhn@mas.ac.mn (B.N.); gantulgad@mas.ac.mn (G.D.); 5 Department of Molecular Biology and Genetics, School of Bio-Medicine, Mongolian National University of Medical Sciences, Ulaanbaatar 14210, Mongolia; munkhtsetseg.ba@mnums.edu.mn; 6 Italian Institute for Environmental Protection and Research (ISPRA), Via Brancati 48, 00144 Rome, Italy; francesco.riga@isprambiente.it; 7 Wildlife Initiative, Bayangol District, Ulaanbaatar 210349, Mongolia; claudiocites@gmail.com; 8 Natural History Museum, Botanical Collections “Filippo Parlatore”—Via Giorgio la Pira 4, 50121 Florence, Italy; l.cecchi@unifi.it

**Keywords:** soil desert, soil microorganisms, bacterial communities, microbiome

## Abstract

Understanding how microbial communities survive in extreme environmental pressure is critical for interpreting ecological patterns and microbial diversity. Great Gobi A Strictly Protected Area represents an intriguing model for studying the bacterial community since it is a protected and intact wild area of the Mongolian desert. In this work, the composition of a bacterial community of the soil from four oases was characterized by extracting total DNA and sequencing through the Illumina NovaSeq platform. In addition, the soil’s chemical and physical properties were determined, and their influence on shaping the microbial communities was evaluated. The results showed a high variability of bacterial composition among oases. Moreover, combining specific chemical and physical parameters significantly shapes the bacterial community among oases. Data obtained suggested that the oases were highly variable in physiochemical parameters and bacterial communities despite the similar extreme climate conditions. Moreover, core functional microbiome were constituted by aerobic chemoheterotrophy and chemoheterotrophy, mainly contributed by the most abundant bacteria, such as Actinobacteriota, Pseudomonadota, and Firmicutes. This result supposes a metabolic flexibility for sustaining life in deserts. Furthermore, as the inhabitants of the extreme regions are likely to produce new chemical compounds, isolation of key taxa is thus encouraged.

## 1. Introduction

The Gobi Desert, the largest Asian desert, is located across southern Mongolia and it is considered one of the world’s largest and most intact remaining wild areas. The term “Gobi”, literally meaning “the waterless place” in the Mongolian Language, indicates a region characterized by extremely continental and dry climate, with long cold winters and short hot summers [1]. The temperature ranges from −40 °C in winter to +40 °C in summer; the average precipitation varies significantly across this region, from scarce in the west (50 mm) to considerably intense in the northeast (200 mm) [2,3]. This extremely harsh environment reflects a unique ecosystem that provides a critical habitat for various rare species of flora and fauna.

For these reasons, according to the Mongolian Law on Special Protected Areas, the Great Gobi is a Strictly Protected Area (SPA), i.e., an “area that can represent unique features and characteristics of natural zones, have preserved its original conditions, and is of special scientific and cultural significance” [4]. It is, in turn, divided into two ecologically distinct parts, the Trans-Altai Gobi (Gobi “A”) and the Dzungarian Gobi (Gobi “B”), which are separated by around 300 km from each other and represent one of the largest protected areas worldwide [5].

Great Gobi A was also included in the World Network of Biosphere Reserves in 1991 as one of the largest biosphere reserves in the world. The goal is to conserve and protect biodiversity, soil, and water resources, to assist in maintaining traditional ways of life, and to increase local participation in the biosphere reserve management [4,5]. Notably, more than 50 oases, which serve as critical water sources for biodiversity, have been documented within the SPA [3].

The Gobi Desert, together with Atacama, Sahara, and Namib deserts, is classified as an extreme desertic environment. The living conditions at the surface of these areas are a challenge for microorganisms, as there is little available water and nutrients, a very large range of temperatures, and high exposure to UV irradiation from the sun. However, despite their inhospitable physiochemical conditions, these habitats provide diverse ecological niches for a wide range of microorganisms from all three domains of life, adapted to thrive in these extreme environments [6,7], playing a key role in the regulation and maintenance of the essential ecosystem services in such habitats [8,9]. Studies report that in these extreme habitats, most microorganisms exist as consortia that provide robustness and extensive metabolic capabilities, enabling them to establish important relationships [10,11]. However, while many studies have addressed the impact of environmental conditions on plant growth and their physiological responses, desert soils are still an unexplored environment from a microbiological viewpoint, since studies about soil microbial communities in arid ecosystems and their metabolic potential are extremely scarce. Some of the few available examples of investigation of the total bacterial community of Gobi Desert soils was provided by An and co-workers, who reported the composition of the bacterial community of a couple of samples from the top of dunes, dominated by Firmicutes, Proteobacteria, Bacteroidetes and Actinobacteriota phyla [12]. Such results are mostly in accordance with those from other studies carried out in the Atacama Desert in Chile, the desert–oasis ecosystem of Shiyang River Basin in China the Central Negev Desert in Israel, the outskirts of Roxby Downs in South and of the culturable bacterial communities from Jordan and Morocco deserts, which reported an unexpected high bacterial diversity in such extreme environments [13,14,15,16,17,18]. However, a more in-depth description of the bacterial community composition and structure of these deserts has not yet been reported and little is known about the interrelations existing between microbial activity and soil physical parameters in desert lands [19]. Moreover, such results are mostly obtained by studies carried out on samples collected from sandy dunes, with high temperatures and soil nutrient limitation [20]. On the other hand, desert oases represent a research hotspot in ecology and biodiversity, where much endemic plant, animal and microbial biodiversity coexists and responds well to environmental changes and stress conditions occurring in such extreme areas such as drought [21]. Such unique environments are quite fragile and complex, and are often the product of several isolation, adaptation and integration events through time and space, where cultural and natural factors influenced the development and interconnection among oases [22]. Nevertheless, the changes in soil properties could directly affect the absorption of water and nutrients by plants as well as the structural and functional diversity of the soil microbiota.

In this context, considering the effects of land-use management or other human activities could have on biodiversity conservation in desert oases at different scales, Great Gobi A provides an interesting model to investigate the microbial biodiversity of a wild and extreme environment, scarcely influenced by the human presence [23]. The aim of the present work was to characterize the total bacterial community from soil samples collected in four oases located in the Great Gobi A SPA, where most of the soil biodiversity is expected. To the best of our knowledge, to date, research in this area has predominantly focused on vegetation and animals. Still, there are no studies in which the soil bacterial communities of Great Gobi “A” were characterized. In our opinion, this work could be a springboard for further research in this field, aiming to fill some of the current gaps in the knowledge and understanding of the bacterial adaptations to desert environments.

## 2. Materials and Methods

### 2.1. Study Area and Oases Soil Sampling

**Figure 1:**
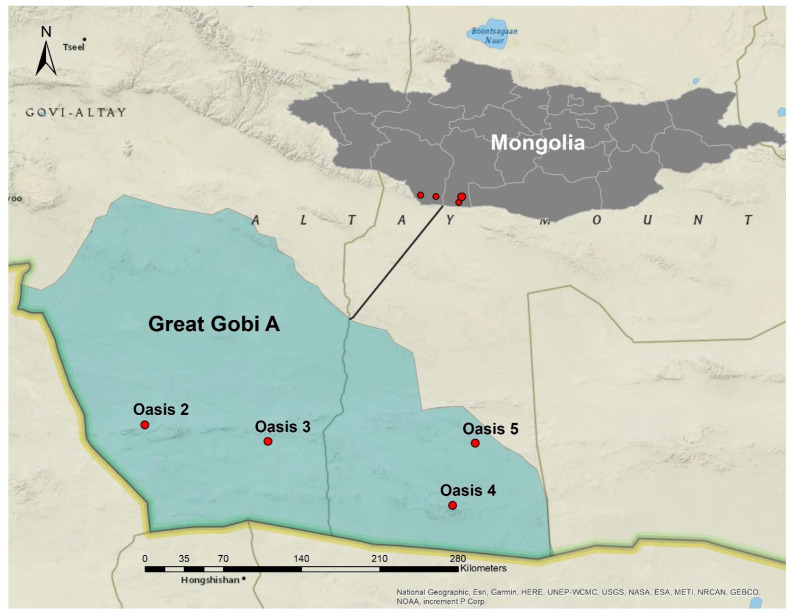
The Great Gobi A Strictly Protected Area’s geographic position, is in blue, and Oases are in red dots (Oasis 2: LAT 43.35308333 LONG 96.34411667; Oasis 3: LAT 43.30285000 LONG 97.77906667; Oasis 4: LAT 42.88171667 LONG 98.81793333; Oasis 5: LAT 43.24652002 LONG 99.00125125).

The sampling was performed in the Great Gobi A Strictly Protected Area, which is located in the southwest part of Mongolia and covers an area of 4.419 million hectares; the elevation ranges from 525 to 2683 m a.s.l. (Figure 1). It is one of the most arid areas in central Asia. Annual precipitation ranges from 30 to 140 mm [24] concentrated from July to August and temperature ranges from −34 °C to 40 °C.

From a phytogeographical viewpoint, this area belongs to the Mongolian Province (Holarctic Kingdom, Tethyan Subkingdom, Irano-Turanian Region, Central Asiatic Subregion) according to Takhtajan and corresponds to the Trans-Altai Gobi floristic province as described by Grubov, an area with very low precipitation and salt-enriched soil [25,26]. Consequently, it is among those with the lower floristic diversity, with only 356 registered species of vascular flora, among which two (*Cleome gobica* Grubov and *Saussurea gubanovii* Kamelin) are endemic and other two (*Leymus ordensis* Peschkova and *Saussurea catharinae* Lipsch.) subendemic [27]. Main perennial, halophytic vegetation in the desert, when present, is made up of scattered saxaul (*Haloxylon ammodendron* (C.A.Mey.) Bunge) or other Caryophyllales (such as and *Reaumuria soongarica* (Pall.) Maxim.) shrubs, often enriched and locally characterized by co-dominant species of *Anabasis*, *Artemisia*, *Calligonum*, *Ephedra*, *Nitraria* and *Zygophyllum*. Wadis and oasis created by isolated springs are very common, as it is the case of those concerned by the present study, sharply marked by a mixed *Phragmites australis* (Cav.) Trin. ex Steud., *Populus euphratica* Olivier and *Tamarix spp*. vegetation, with significant presence of representatives of *Artemisia*, *Atraphaxis*, *Lycium* and others, and with very localized floristic variations along the gradient that marks the ecotonic environment between the center of the oasis and its margins.

In the Trans-Altai Gobi region, since wells and open water sources are very rare, this is quite important. Most of the territory is protected from anthropo-zoogenic effects by its extremely dry environment. The research area is unique from other southern Mongolian regions due to its absence of human influence [28].

Sampling was performed in May 2022 in four oases. Topsoil samples were randomly collected from 5 sampling points into 50 mL sterile falcon (30 × 115 mm) tubes for each oasis. Samples were identified with a progressive number referring to the oasis (from MS2 to MS5) and an additional number (ranging from 1 to 5) indicating the different sampling points. The permission for enter to the Great Gobi A SPA is reported in Supplementary Figure S1.

### 2.2. Chemical and Physical Characterisation of the Soil Samples

In total, 10–20 g of soil samples were air-dried and sieved through a 2 mm mesh. For C and N quantitative analysis and C chemical fractionation, soil sub-samples were ground and homogenized to 0.5 mm. Total carbon (TC), composed of mineral and organic carbon, and total nitrogen (TN) contents in the bulk soil were measured by dry combustion on a Thermo Flash 2000 CN soil analyzer through the Eager Experience for Flash Elemental Analyzer, as reported by Valboa et al. (2015) [29]. To achieve this, 15–20 mg of soil were weighed into Ag-foil capsules and analyzed for the TC and TN. Other 35–40 mg of soil were pre-treated with 10% HCl until complete removal of carbonates and measured the total organic carbon (TOC). After that, the total mineral carbon, expressed by equivalent calcium carbonate (CaCO_3_), was determined, calculated from the difference between the total carbon and organic carbon and multiplied by coefficient 8.333 [30]. Soil pH was measured on a 1:2.5 soil–water suspension with a Metrohm 654 pH meter. Moreover, the particle size distribution was analyzed by the Sedigraph (Micromeritics Instrument Corp., Norcross, GA, USA) apparatus which is based on the sedimentation method. Sample preparation and analytical procedure followed the suggestion of Andrenelli et al. (2013) [31]. Each sample was sieved at 2 mm; from each sample, 5 g was taken and used to obtain a soil suspension passed through a 250 μm wet sieve to detect medium, coarse, and very coarse sands. All soil suspensions were replicated three times and automatically loaded by Mastertech auto-sampler. A solution of Calgon (0.2%) in sucrose (50%) was prepared to analyze the curve between 50 and 250 μm, assuring conformity to Stokes’ law. The initial part of the curve was analyzed by Sedigraph, starting from a soil suspension passed through 250 μm but adopting a solution of Calgon (0.2%) in distilled water to reduce the occurrence of Brownian motions. To obtain an accurate solution of Stokes’ law, particle density was measured for each sample using a helium pycnometer. The device has software for data acquisition and automatic data analysis. The measurements were replicated three times for each sample.

### 2.3. Extraction of Total DNA and Next-Generation Sequencing

DNA extraction was performed from 250 mg of each soil sample using the PowerLyzer^®^ PowerSoil^®^ DNA Isolation Kit (Qiagen, Hilden, Germany) following the manual instructions. The quality and quantity of the extracted DNA were checked by 0.8% agarose gel electrophoresis and Lite Plus NanoDrop spectrophotometer (Thermo Fisher Scientific, Waltham, MA, USA). Hypervariable regions of the 16S rRNA (V1–V9) were used as molecular markers to identify bacterial taxa [32]. In this work, the V3–V4 regions were amplified via PCR using primers 338 F: ACTCCTACGGGAGGCAGCA and 806 R: GGACTACHVGGGTWTCTAAT, and amplicons were purified, quantified, and homogenized to obtain sequencing libraries [33]. Then, libraries were sequenced on Illumina Novaseq 6000. PCR amplification, library construction, and sequencing were performed by an external company (BMKGENE, Beijing, China). Sequence files were submitted to the NCBI sequence read archive (SRA) and are available under accession PRJNA1056917.

### 2.4. Sequence Analysis

PCR primers were removed from all the sequences using Cutadapt (version 3.5) with a maximum error rate of 0.15 [34]. Then, the partial 16S rRNA gene sequences were clustered into ASVs (amplicon sequence variants) following the DADA2 pipeline (version 1.16) (described at https://benjjneb.github.io/dada2/tutorial.html, accessed on 30 May 2023) [35] using the R software version 4.2.3 [36]. For filter and trimming, parameters “truncLen = 0” and “truncQ = 10” were used, while for the dada algorithm, the pool = “pseudo” option was used. The taxonomic annotation was performed using the DECIPHER R package [37] on the Silva database version 138 [38]. Following developer instructions, the functional traits database FAPROTAX [39] was used to associate potential functions to bacterial ASVs based on their taxonomy.

### 2.5. Statistical Analysis

All statistical analyses were performed in the R environment, version 4.3.1. Chemical and physical features were analyzed using Principal component Analysis (PCA) and HCPC in the FactoMineR package (version 2.8).

Bacterial diversity was estimated using the microbiome packages (version 1.22.0). The function “alpha” of microbiome packages was used to compute the Shannon index, evenness and observed richness. Pearson correlation analysis between alpha diversity indices and physiochemical parameters was performed using the “corr.test” function with a psych package. Differences in bacterial diversity between the different oases were tested using the Kruskal–Wallis non-parametric test.

Beta-diversity was analyzed with the Bray–Curtis dissimilarity, using the “transform_sample_counts” and “ordinate” functions of the phyloseq package.

Different community structures were tested using permutational multivariate analysis of variance (“adonis2” function of the vegan R package) with 999 permutations.

The functional prediction of soil microbiome obtained by the FAPROTAX database was visualized by heatmap using pheatmap function of pheatmap package (version 1.0.12).

## 3. Results

### 3.1. Characterization of Soil Physicochemical Features

The analysis of the different soil particle content (sand, clay, silt) showed that the oases are characterized by different soil texture classes (Table 1). More specifically, samples of Oasis 2 showed a prevalent sand texture with the highest mean sand content (86.18%) (Table 2). This sand content decreases moving from Oasis 2 to Oasis 5, which is characterized by a loam texture and the highest silt level. However, within each oasis, there is variability in the soil particle content, as reported by the calculated coefficients of variation (CV) (Table 2). For example, samples from Oases 2 and 3 are composed predominantly of sand (on average higher than 80%), with a similar silt content (12%) and low levels of clay. Still, the variations in silt content (CV = 77.92%) among samples of Oasis 2 resulted in sand and loamy sand textures. In addition, in Sample 5 of Oasis 3 (Sample MS3_5), the clay amount is 12 times higher than that of the other samples of the same oasis, showing a sandy loam texture. On the other hand, samples of Oases 4 and 5 are composed of a lower amount of sand and a higher amount of silt and clay, around two-fold higher than that of the other two oases, determining a loamy sand and sandy loam texture, respectively. Samples of Oasis 5 are characterized by the lowest amount of sand (on average 36.99%) and the highest amount of silt (45.42%), thus with loam and silt-loam textures, with the only exception of MS5_5, which is characterized by sandy loam texture due to the two-fold higher amount of sand and the two-fold lower amount of silt particles compared to the other samples.

Regarding soil chemical parameters (Table 1), data obtained showed an alkaline pH for all samples that ranged from minimum values in Oasis 2 (on average 7.95) to the highest values in Oasis 4 (mean value 8.75). Oasis 2 samples are characterized by the absence or scarcity of total nitrogen (0%) and organic carbon (0.15%), according to the predominant sand profile textures. These parameters increase from Oasis 3 to a maximum in Oasis 5, per loam textures. Also, calcium carbonate (CaCO_3_) content seems to be positively correlated to pH level per oasis: a low concentration of organic carbon and a high concentration of CaCO_3_ result in soils with a significantly alkaline pH. As shown in Table 1, the CaCO_3_ has a similar concentration between Oases 4 and 5. Still, the pH of Oasis 4 is higher than that of Oasis 5 due to lower concentrations of organic carbon in Oasis 4 (mean value = 1.64%) than in Oasis 5 (4.05%). TOC values for two samples (MS4_2 and MS5_1) are significantly higher than those of the other samples, highlighting that these were collected in peat environments. Due to the diversity of these two soils, they were discarded from all subsequent analyses. 

Results of univariate analysis of variance (one-way ANOVA) showed that the average values of soil properties significantly differ among oases (Table 2). The post hoc test indicates a significant difference between Oases 2 and 4 for the TC and CaCO_3_ values, with the lowest content of these parameters in Oasis 2 and the highest in Oasis 4. In addition, the amount of sand, clay, and silt is significantly different in Oasis 5 compared to the other oases.

**Figure 2:**
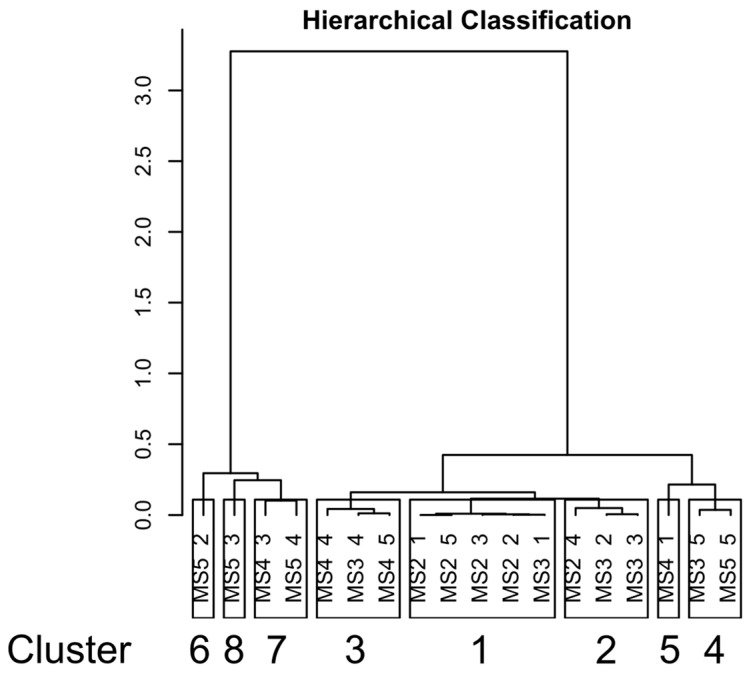
Results of the Hierarchical Clustering Analysis (HCPC) performed on soil samples based on the chemical and physical properties which resulted significantly different among the oases. The number of clusters below each cluster is relative to performed hierarchical clustering.

The soil physicochemical variables that resulted in significant differences among the oasis were used in multivariate analysis. Principal component analysis (PCA) and hierarchical clustering analysis (HCPC) were used to explore the samples’ similarities further and identify potential soil clusters based on chemical and physical properties. The results (Figure 2) highlighted the presence of eight clusters composed of samples belonging to different oases, except for four out of the five samples of Oasis 2 that grouped together. Moreover, three clusters were formed of only one sample. The top drivers of cluster formation were assessed by v-test and *p*-value, reported in Table 3. As reported by these tables, in Cluster 1, the sand variable was more representative than silt and TC, which were less representative. Instead, the clay, silt, and TC variables were more representative in Clusters 6 and 7. Cluster 8 was only explained by CaCO_3_. 

### 3.2. Characterization of Soil Bacterial Communities

**Figure 3:**
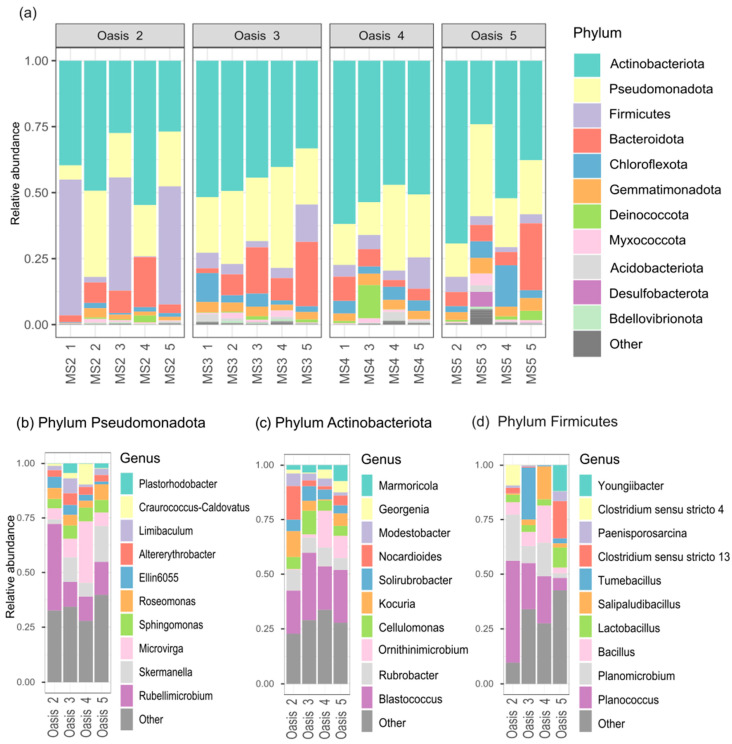
(**a**) Taxonomic composition at the phylum level of the soil samples, separated based on the oasis of origin. Phyla with reads frequency lower than the 1% of the whole community were reported as “Other”. Taxonomic composition of the 10 predominant genera of oasis belonging to (**b**) Pseudomonadota, (**c**) Actinobacteriota, and (**d**) Firmicutes Phylum. Genera that are not present in the first 10 were reported as “Other”.

The soil bacterial communities of the topsoil samples from the four oases were characterized by NGS, generating 8,315,430 pairs of raw reads. These reads were processed for quality control, assembly, and data filtration, which yielded 3,715,854 clean reads. A minimum of 70,902 clean reads were generated for each sample, and the average data output per sample was 288,563 clean reads. The clean sequences were clustered into 32,437 ASVs (see Section 2). The taxonomic analysis highlighted 47 bacterial phyla, with 11 showing a relative abundance greater than 1% of the total reads (Figure 3a). The phyla that are most represented in all the oases were Actinobacteriota (44.2%), Pseudomonadota (23.6%), and Firmicutes (8.83%), with a different degree of abundance between the oases. Actinobacteriota was the dominant phylum, constituting 39%, 44%, 53%, and 42% of the reads for Oases 2, 3, 4, and 5, respectively, followed by Pseudomonadota and Firmicutes. Within the Oasis, the abundance of reads varied among the samples. In particular, the abundance of reads assigned to Actinobacteriota was more similar in Oases 3 and 4 than in Oases 2 and 5. Instead, the Firmicutes phylum showed the highest abundance of reads in MS2_1 (51.5%), MS2_3 (42.9%), and MS2_5 (44.8%) samples of Oasis 2 (Figure 3a).

At the class level, the Pseudomonadota phylum was mostly constituted by AlphaProteobacteria (8.49 × 10^−1^) followed by GammaProteobacteria (1.51 × 10^−1^). More in detail, the Alphaproteobacteria classes showed a similar distribution of abundance in all samples of Oases 2, 3, 4 and Oasis 5, except for Sample 3 of Oasis 5, which was composed of the highest abundance of GammaProteobacteria. In addition, we considered the 10 most predominant genera of the three representative phyla (Figure 3b–d). Most of the reads of the Pseudomonadota phylum were mainly classified in *Rubellimicrobium*, *Microvirga* and *Skermanella* genus with a significant difference of abundance of *Rubellimicrobium* in Oasis 2 compared to other oases (Figure 3b). The distribution of actinobacterial community at the genus level was mostly homogeneous between the oases; among these, the most abundant genera were *Blastococcus* followed by *Rubrobacter* (Figure 3c). Lastly, in the Firmicutes phylum, most of reads were classified in Bacilli classes followed by Clostridia ones, with different member of genera. In particular, in Oasis 2, there were three samples in which the highest abundance of reads was detected; the most abundant genera were *Planococcus* (Caryophanaceae family) and *Planomicrobium* (Planococcaceae family) of Bacilli classes. Furthermore, a great abundance of different member of genera belonging to Bacillaceae family (*Bacillus*, *Tumebacillus*, *Lactobacillus*, and *Salipaludibacillus*) in Oases 3 and 4 was found. Instead, a prevalent presence of the Clostridiaceae family in Oasis 5 was detected (Figure 3d).

**Figure 4:**
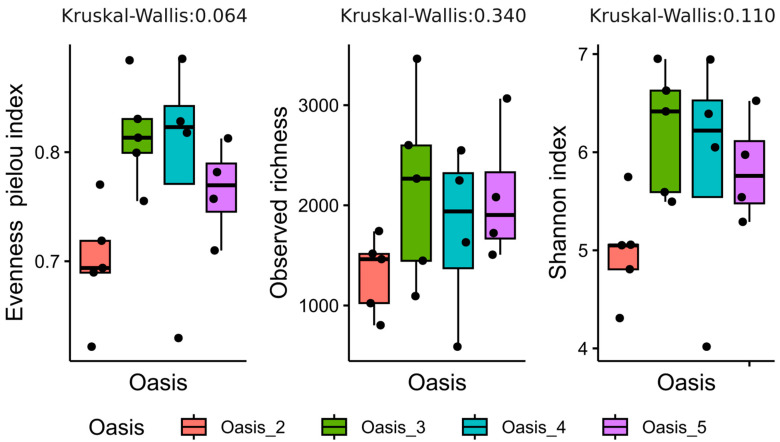
Indices of the diversity of the soil bacterial community of the different oases from the Great Gobi A desert. The observed richness, Shannon diversity indices, and evenness indices are reported. The alpha diversity indices of each sample within oasis along the boxplot are indicated by dots.

Furthermore, we explored alpha diversity by calculating three diversity indices: the Pielou evenness index, observed richness and the Shannon index (Figure 4). Results suggest that samples belonging to Oasis 2 generally showed lower alpha diversity values than those in other oases. In particular, lower richness, evenness, and Shannon index values were observed than in other oases, which showed a similar index (Figure 4). The alpha diversity indexes were not significantly different between oases, as demonstrated by Kruskal–Wallis’s test (*p*-value  >  0.05). Pearson correlation showed that the alpha diversity was not statistically related to chemical and physical parameters.

### 3.3. Beta Diversity Analysis

**Figure 5:**
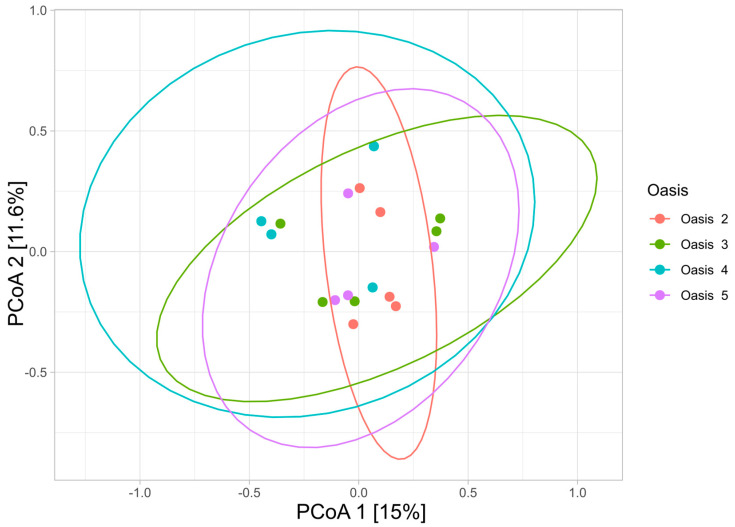
Principal coordinate analysis (PCoA) based on the unweighted Bray–Curtis microbiome metric among all samples. The percentages of variation explained by PC1 and PC2 are indicated in the axis.

Principal Coordinate Analysis (PCoA) was performed using the Bray–Curtis distance at the ASV level to compare the bacterial communities of the different oases. The results obtained revealed an evident clustering of samples independently from the oasis of origin (Figure 5).

Despite the absence of evident clustering in ordination analysis, PERMANOVA analysis reported a statistically significant effect of the “oasis” variable on the sample’s bacterial composition (R^2^ = 22%, *p*-value = 0.03). 

In addition, the effect of the clustering obtained by HCPC analysis on soil physiochemical variables was tested on the soil bacterial communities, showing a high contribution in explaining the bacterial variability among samples (R^2^ = 47%, *p*-value = 0.012).

### 3.4. Functional Potential of Soil Microbiomes

**Figure 6:**
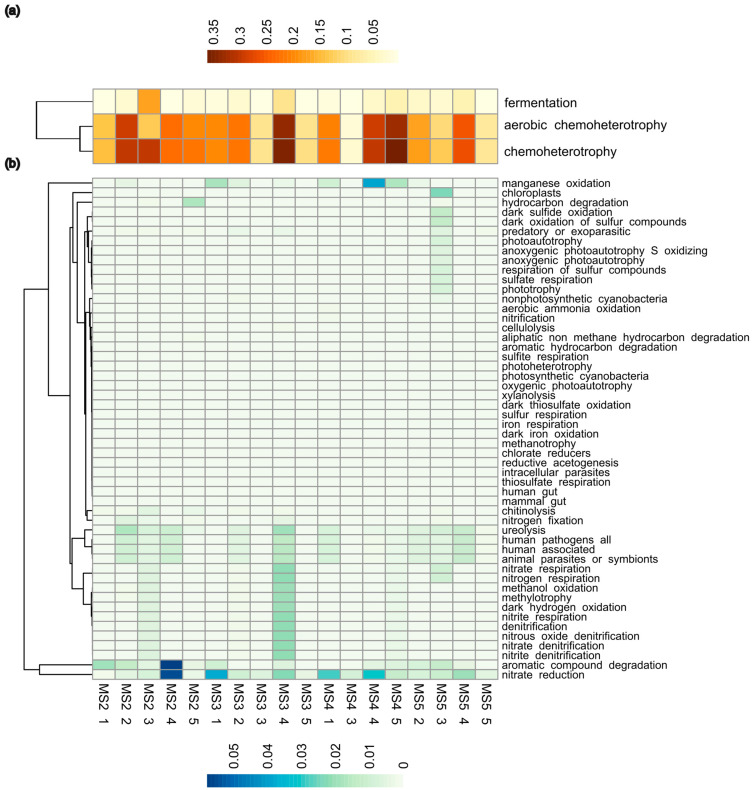
Heatmap showing the relative abundance of reads associated with functions predicted by FAPROTAX. The results are divided into high abundant (**a**) and low abundant (**b**) based on 1% of threshold level of median relative abundance.

The soil bacterial putative functions in desert ecosystems were predicted using FAPROTAX to investigate a pattern of potential functions specifically connected to the different oases. Data obtained were explored by a heatmap. To better visualize the results, the functional bacterial community was divided into high-abundant (Figure 6a) and low-abundant (Figure 6b) portions based on median relative abundance (1% threshold). The core bacterial functional group was constituted by methanol oxidation, methylotrophy, dark hydrogen oxidation, manganese oxidation, fermentation, aerobic chemoheterotrophy, predatory or exparasitic and chemoheterotrophy functions, which represent only 14% of the total functional group identified. Among those functions, chemoheterotrophy (2.07 × 10^−1^), followed by aerobic chemoheterotrophy (1.88 × 10^−1^) and fermentation (3.39 × 10^−2^), showed a higher mean relative abundance of reads, as visualized by a heatmap (Figure 6a). The more representative phyla associated with ASV of these predictive functions were mainly Actinobacteriota, Pseudomonadota, Bacteroidota, and Firmicutes. Considering the functional bacterial community with read abundance lower than 1% (Figure 6b), the heatmap highlighted the presence of a nitrate reduction function in most of the samples. Moreover, Sample MS3_4 reported a specific higher abundance of reads associated with the nitrogen cycles, such as nitrate, nitrite denitrification, and nitrite respiration; on the other hand, a higher abundance of reads in Sample MS5_3 was correlated with sulfur cycling and other functions related to extreme environments, such as anoxygenic phototrophy.

## 4. Discussion and Conclusions

Characterizing bacterial communities in extreme environments is essential to further research, with many applications in different fields. Thus, studying the Great Gobi A Strictly Protected Area is increasingly interesting for understanding bacterial diversity since it is a protected area combined with extreme climate conditions.

Based on chemical and physical features, the four oases significantly differed in the soil texture profile, total carbon (TC) content, and calcium carbonate (CaCO_3_). These parameters varied from those of Oasis 2, with the lowest levels of TC and CaCO_3_ and dominant sandy texture, to Oases 4 and 5, exhibiting higher levels of TC and CaCO_3_ and loam texture. However, a high variability of these parameters was observed within each oasis, especially Oasis 4 for soil textures and Oasis 5 for the TC and CaCO_3_. The clustering of samples based on the physiochemical variables showed that only samples belonging to Oasis 2, which were grouped, were characterized by similar properties. In contrast, the variability in samples from Oases 4 and 5 resulted in clusters of samples of different oases. Considering that these soil samples were collected at different points within each oasis, the results of this characterization showed that Oasis 2 was more homogeneous and poorer in nutrients, suggesting an arid and non-anthropized environment; this is in accordance with the observations of Delgado-Baquerizo et al., who reported a negative correlation between aridity and availability of carbon and nitrogen [40]. Instead, the other oases were characterized by higher diversity in terms of physiochemical properties, with samples composed of higher amounts of total carbon and calcium carbonate than other samples of the same oasis, suggesting a high diversity in terms of physiochemical properties, probably due to the presence of various vegetation in some sampling points. 

Concerning the microbiological analysis of soil samples, the analysis of total bacterial communities, obtained by NGS analysis on bacterial 16S rRNA genes, revealed a lower biodiversity of samples from Oasis 2 compared to the other oases, which showed a similar biodiversity in terms of evenness, richness and Shannon index (Figure 4). This result was in accordance with the physiochemical properties of Oasis 2. Indeed, a higher aridity and a lower availability of nutrients might presumably affect soil biodiversity. 

The phyla Actinobacteriota, Pseudomonadota and Firmicutes accounted for most of the bacterial reads, in agreement with previous data, as reported in studies across Namib, Taklamaken deserts (China), the Atacama Desert and other deserts [12,41,42,43,44]. Nevertheless, the abundance of ASV related to members of genera was diverse between samples of the oases and when comparing other deserts. This diversity is also supported by a study conducted across other areas of Gobi Desert, reporting a significantly difference between samples taken at a distance of 5 km [42].

It is not surprising that few members of the Acidobacteria were observed, likely due to the alkaline pH of these samples. Interestingly, the Actinobacteriota and Firmicutes were dominant across the four oases, confirming their adaptation to similar environments [20,41,45,46]. Such adaptation is likely due to the high G+C content tolerance to UV radiations for Actinobacteriota, and endospore-forming Firmicutes, enabling them to survive in challenging dry conditions [41,45,46]. Actinobacteriota dominates all oases, excluding three samples of Oasis 2, where Firmicutes dominates. A possible reason is that Actinobacteriota seem to colonize the finer particle fraction and silt and clay content better. Despite incomplete information on the linkage between soil texture and bacterial community composition, all these studies support the presence of texture-sensitive/responsive taxa [47].

On the other hand, the mechanisms underlying the adaptation to desiccation of most Pseudomonadota remain to be clarified. Nevertheless, Pseudomonadota dominate more in Oases 2 and 3, where nutrients are lower. In general, Pseudomonadota includes many genera, capable of nitrogen fixation and growing at low carbon or nitrogen concentrations, making them ideally suited to this low C and N habitat [48]. That is not surprising; in fact, the most abundant genera found in all oases were *Microvirga*, which was related to nitrogen fixation in different studies, and *Rubellimicrobium,* which was generally retrieved from sand soil, where nutrients are lower [49,50,51].

The evaluation of the community structure through PCoA ordination analysis reported a separation of samples independently from oases. This could suggest that the distribution of samples is related to the combination of specific physiochemical variables. Indeed, the PERMANOVA test performed on the clusters of physiochemical variables showed the highest contribution in separating the samples.

Considering the high variability of bacterial communities within the Oasis, we investigated whether the bacterial community functions were more conserved within the different oases. We used the FAPROTAX tool for the prediction of functions. The results showed that the core bacterial functional group identified among all samples of different oases represents only 14% of the total putative functions identified. The functional groups with the highest abundances were aerobic chemoheterotrophy and chemoheterotrophy, mainly contributed by the most abundant bacteria, such as Actinobacteriota, Pseudomonadota, Bacteroidota, and Firmicutes [52]. The abundance of chemoheterotrophy in desert soil suggests that many microbes, such as photoautotrophic bacteria, despite their potential of carbon fixation, are limited in dry desert, while heterotrophic bacteria have a flexible metabolic ability with different strategies in sustaining life in deserts and other extreme soil environments [16,53]. Despite the core bacterial function being represented by few functions, the PERMANOVA analysis confirms there is no separation of functions per oasis.

In addition, two samples belonging to Oasis 2 (MS2_3) and 3 (MS3_4) contain bacteria that may be denitrifiers due to a higher abundance of ASV associated with the *Paracoccus* genus of Pseudomonadota phylum, which has been linked with in nitrate and nitrite denitrification processes. Despite the fact that it is commonly known that this function occurs in moist soil, denitrifying organisms can survive in harsh conditions [54]. Studies carried out in the Atacama Desert have shown the presence of genes related to denitrification. In particular, the authors showed that after incubation of the soil with water, nitrate, and glucose-C, denitrification-related genes capable of transforming NO^3−^ to N_2_ were detected in all incubated soils. Their functional significance was also confirmed by the observed measurements of N_2_O and N_2_ production [54]. Furthermore, a higher abundance of reads found is associated with anoxygenic phototrophs bacteria, a kind of extremophile bacteria that thrive in habitats characterized by extremes of temperature, pH, or salinity. In conclusion, the data obtained showed that, despite the different origins, the samples of different oases grouped. Data obtained suggested that the oases were highly variable in physiochemical parameters and bacterial communities despite the similar extreme climate conditions.

## 5. Conclusions

To conclude, the results of characterization of this protected area highlighted how, although there are extreme climatic conditions, there is a high diversity of bacterial communities both on short and long distances. These desert habitats are especially rich in Actinobacteriota and Firmicutes as well as Pseudomonadota, which are well-adapted to survive in these resource-poor environments. Metabolic flexibility and inorganic energy sources are important in sustaining life in deserts and other extreme soil environments. In addition, Actinobacteriota are highly attractive because of their unparalleled ability to synthesize a wide range of natural chemicals with various bioactivities. It is also believed that actinobacteriota populations that are diversified and found in severe environments are more likely to create new chemical entities. Isolation of the natural habitats in such extreme areas and designing improved procedures for selective isolation of key taxa is thus encouraged, as the inhabitants of the extreme regions are likely to produce new chemical compounds. Moreover, the study of these desert microorganisms may aid in efforts to mitigate and prevent the spread of deserts and/or to restore soil/vegetation cover and also offer the opportunity to discover novel organisms or biomolecules such as new thermostable or alkaline-stable enzymes [55].

## Figures and Tables

**Table 1:** Physiochemical parameters associated with soil samples belonging to the different oases.

	Oasis	pH	TC %	TOC %	TN %	C/N	Tot CaCO_3_ %	Sand	Clay	Silt	Texture Profile	Temperature (°C)	Precipitation (mm)
MS2_1	Oasis_2	NA	0.46	0.06	0.00	NA	3.30	91.21	2.97	5.83	sand	23.64	1.40
MS2_2	Oasis_2	7.85	0.47	0.24	0.00	NA	1.90	91.61	1.00	7.39	sand	23.64	1.40
MS2_3	Oasis_2	8.2	0.37	0.08	0.00	NA	2.40	86.22	3.03	10.75	loamy_sand	23.64	1.40
MS2_4	Oasis_2	NA	0.44	0.23	0.00	NA	1.80	71.37	1.00	27.63	loamy_sand	23.64	1.40
MS2_5	Oasis_2	7.8	0.52	0.15	0.00	NA	3.10	90.48	2.70	6.83	sand	23.64	1.40
MS3_1	Oasis_3	8.6	0.46	0.13	0.00	NA	2.70	89.12	1.00	9.88	sand	25.59	0.50
MS3_2	Oasis_3	7.95	1.17	1.04	0.04	23.30	1.10	82.67	1.00	16.33	loamy_sand	25.59	0.50
MS3_3	Oasis_3	8.29	1.33	1.03	0.07	15.40	2.50	82.96	1.54	15.50	loamy_sand	25.59	0.50
MS3_4	Oasis_3	7.8	1.44	0.98	0.06	17.30	3.80	90.52	1.00	8.48	sand	25.59	0.50
MS3_5	Oasis_3	8.52	0.95	0.36	0.15	2.40	4.90	72.61	12.85	14.54	sandy_loam	25.59	0.50
MS4_1	Oasis_4	8.49	2.88	1.91	0.13	14.90	8.20	67.22	4.19	28.59	sandy_loam	23.07	2.10
MS4_2	Oasis_4	8.12	14.92	9.15	0.76	12.10	48.10	11.42	19.14	69.44	silt_loam	23.07	2.10
MS4_3	Oasis_4	9.82	4.70	3.61	0.24	15.30	9.00	46.30	18.35	35.35	loam	23.07	2.10
MS4_4	Oasis_4	8.3	1.35	0.45	0.03	14.40	7.50	80.25	1.00	18.75	loamy_sand	23.07	2.10
MS4_5	Oasis_4	8.38	1.23	0.59	0.02	33.70	5.40	85.33	0.98	13.69	loamy_sand	23.07	2.10
MS5_1	Oasis_5	NA	16.70	15.73	1.48	10.60	8.20	NA	NA	NA	NA	27.20	0.60
MS5_2	Oasis_5	7.75	1.21	0.60	0.02	35.90	5.10	27.14	23.42	49.45	loam	27.20	0.60
MS5_3	Oasis_5	NA	2.52	0.75	0.04	18.60	14.70	30.76	18.69	50.55	silt_loam	27.20	0.60
MS5_4	Oasis_5	8.26	3.51	2.73	0.26	10.50	6.50	29.80	16.44	53.75	silt_loam	27.20	0.60
MS5_5	Oasis_5	8.82	0.83	0.43	0.01	43.50	3.40	60.27	11.79	27.94	sandy_loam	27.20	0.60

Abbreviations: TC: Total carbon; TOC %: Total organic carbon; TN %: Total nitrogen; C/N: carbon/nitrogen; Tot CaCO_3_: Total calcium carbonate; NA: not available.

**Table 2:** Mean values of soil physicochemical parameters per oasis. Only the parameters that resulted in significantly different results among the oasis using the one-way ANOVA test are reported. Standard deviations (SD) and coefficient of variation (CV) are evidenced. The results of multiple Tuckey post hoc pairwise tests are identified in the column “Sig.” by letters. Values with different letters significantly differ (*p*-value < 0.05).

	TC				Tot CaCO_3_				Sand				Clay				Silt			
	Mean	SD	CV (%)	Sig.	Mean	SD	CV (%)	Sig.	Mean	SD	CV (%)	Sig.	Mean	SD	CV (%)	Sig.	Mean	SD	CV (%)	Sig.
Oasis_2	0.45	0.05	11.82	b	2.50	0.70	28.22	b	86.18	8.55	9.92	a	2.14	1.04	48.96	b	11.68	9.10	77.92	b
Oasis_3	1.07	0.39	36.20	ab	3.02	1.43	47.67	ab	83.57	7.08	8.47	a	3.48	5.24	150.79	b	12.94	3.53	27.27	b
Oasis_4	2.54	1.62	63.96	a	7.51	1.57	20.85	a	69.77	17.40	24.95	a	6.13	8.29	135.18	b	24.09	9.72	40.35	b
Oasis_5	2.02	1.23	60.82	ab	7.42	5.01	67.57	ab	36.99	15.59	42.15	b	17.58	4.83	27.49	a	45.42	11.79	25.97	a

**Table 3:** Results of the HCPC analysis with chemical and physical variables. V-test represents the influence of variables in the cluster composition. *p*-value indicates how each variable can explain the cluster.

Cluster	Variables	V-Test	*p*-Value
1	sand	2.19	0.028
	TC	−2.14	0.033
	silt	−2.36	0.018
6	clay	2.20	0.028
	sand	−1.99	0.047
7	TC	3.31	0.00092
	silt	2.11	0.034
	clay	2.05	0.040
	sand	−2.17	0.030

## Data Availability

All sequences were submitted online. Metagenomic sequences were deposited in the NCBI Sequence Read Archive (SRA) under the accession PRJNA1056917.

## Supplementary Materials

The following supporting information can be downloaded at: https://www.mdpi.com/article/10.3390/microorganisms12020320/s1, Figure S1: permit for access to the Great Gobi A SPA.
