# Supplementary material for: The Great Gobi A Strictly Protected Area: Characterization of Soil Bacterial Communities from Four Oases
Source: Microorganisms. 2024 Feb 3;12(2):320. doi: 10.3390/microorganisms12020320 (PMC10891509; doi:10.3390/microorganisms12020320)
Supplement: Supplementary file 1 [file microorganisms-12-00320-s001.zip › microorganisms-2828885-supplementary.pdf]

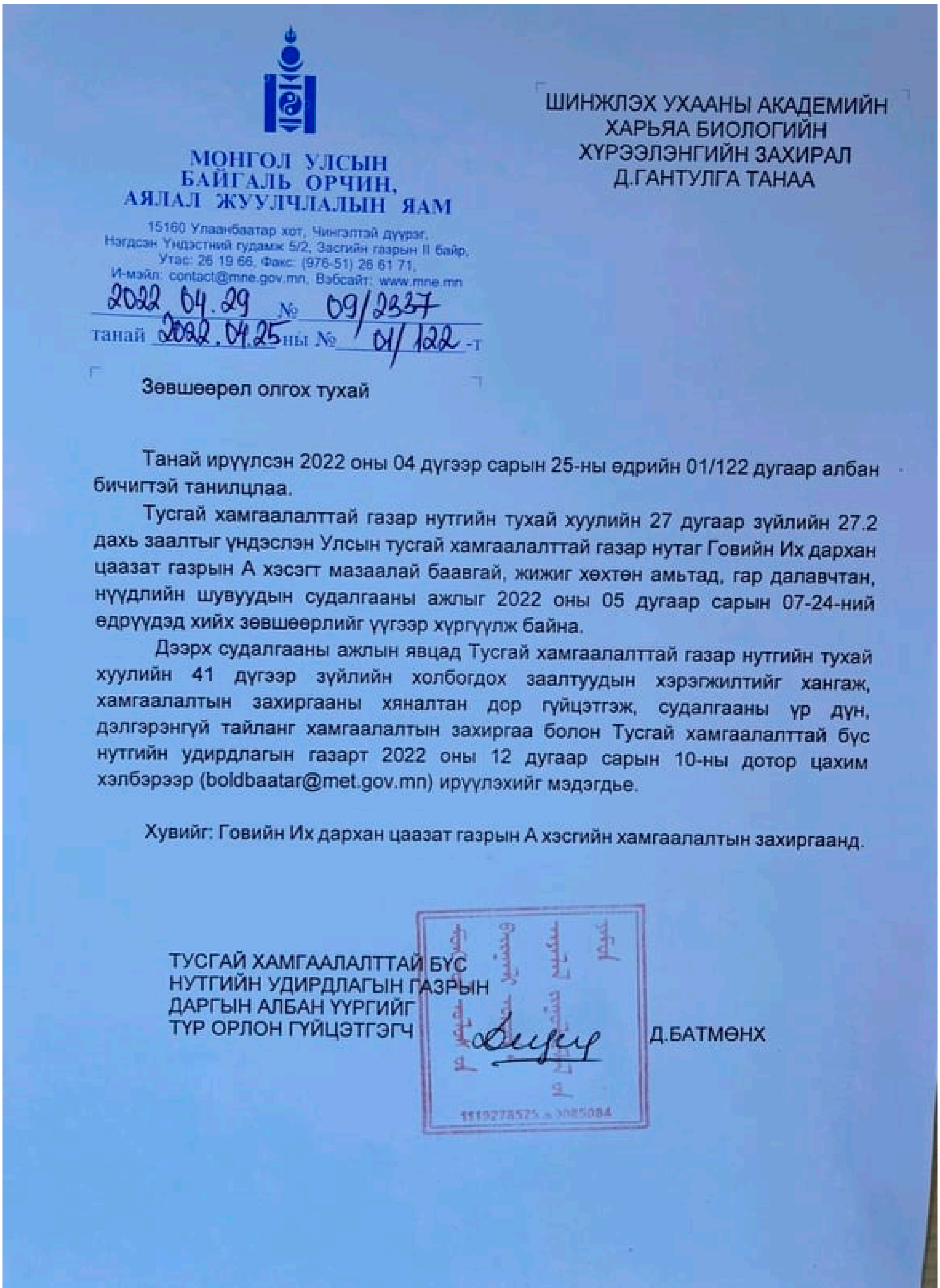

МОНГОЛ УЛСЫН
БАЙГАЛЬ ОРЧИН,
АЯЛАЛ ЖУУЛЧЛАЛЫН ЯАМ

15160 Улаанбаатар хот, Чингэлтэй дүүрэг,
Нэгдсэн Үндэстний гудамж 5/2, Засгийн газрын II байр,
Утас: 26 19 66, Факс: (976-51) 26 61 71,
И-мэйл: contact@mne.gov.mn, Вэбсайт: www.mne.mn

2022.04.29 № 09/2337

танай 2022.04.25-ны № 01/122-т

ШИНЖЛЭХ УХААНЫ АКАДЕМИЙН
ХАРЬЯА БИОЛОГИЙН
ХҮРЭЭЛЭНГИЙН ЗАХИРАЛ
Д.ГАНТУЛГА ТАНАА

Зөвшөөрөл олгох тухай

Танай ирүүлсэн 2022 оны 04 дүгээр сарын 25-ны өдрийн 01/122 дугаар албан бичигтэй танилцлаа.

Тусгай хамгаалалттай газар нутгийн тухай хуулийн 27 дугаар зүйлийн 27.2 дахь заалтыг үндэслэн Улсын тусгай хамгаалалттай газар нутаг Говийн Их дархан цаазат газрын А хэсэгт мазаалай баавгай, жижиг хөхтөн амьтад, гар далавчтан, нүүдлийн шувуудын судалгааны ажлыг 2022 оны 05 дугаар сарын 07-24-ний өдрүүдэд хийх зөвшөөрлийг үүгээр хүргүүлж байна.

Дээрх судалгааны ажлын явцад Тусгай хамгаалалттай газар нутгийн тухай хуулийн 41 дүгээр зүйлийн холбогдох заалтуудын хэрэгжилтийг хангаж, хамгаалалтын захиргааны хяналтан дор гүйцэтгэж, судалгааны үр дүн, дэлгэрэнгүй тайланг хамгаалалтын захиргаа болон Тусгай хамгаалалттай бүс нутгийн удирдлагын газарт 2022 оны 12 дугаар сарын 10-ны дотор цахим хэлбэрээр (boldbaatar@met.gov.mn) ирүүлэхийг мэдэгдье.

Хувийг: Говийн Их дархан цаазат газрын А хэсгийн хамгаалалтын захиргаанд.

ТУСГАЙ ХАМГААЛАЛТТАЙ БҮС
НУТГИЙН УДИРДЛАГЫН ГАЗРЫН
ДАРГЫН АЛБАН ҮҮРГИЙГ
ТҮР ОРЛОН ГҮЙЦЭТГЭГЧ Д.БАТМӨНХ

1119278575 [illegible] 3085084

152273019

Figure S1. permit for access to the Great Gobi A SPA.
